# Biochemical markers and emerging therapies for intestinal manifestation of graft-versus-host disease

**DOI:** 10.3389/fimmu.2025.1645503

**Published:** 2025-09-22

**Authors:** Katarzyna Wiejak, Adam Przybyłkowski, Agnieszka Tomaszewska

**Affiliations:** ^1^ Department of Gastroenterology and Internal Medicine, Medical University of Warsaw, Warsaw, Poland; ^2^ Department of Hematology, Transplantation and Internal Medicine, Medical University of Warsaw, Warsaw, Poland

**Keywords:** graft-versus-host disease, allogeneic hematopoietic cell transplantation, gastrointestinal tract, biomarkers, GVHD treatment

## Abstract

Graft-versus-host disease (GVHD) remains a potentially fatal complication of allogeneic hematopoietic stem cell transplantation (alloHCT). Gastrointestinal involvement, whether in acute or chronic GVHD, is associated with a poorer prognosis and poses significant diagnostic and therapeutic challenges. Numerous studies have attempted to identify markers that could facilitate the diagnosis and predict the course of GVHD. Among them, REG3α and ST2 currently show the greatest promise; however, comprehensive validation remains lacking. Although several new drugs have been approved for GVHD treatment in recent years and some modifications to GVHD prophylaxis have been adopted into clinical practice, further research are needed to validate biomarkers and explore new therapeutic targets, particularly in gastrointestinal GVHD. The review aims to summarize current research on GVHD biomarkers and emerging treatment targets, with a particular focus on gastrointestinal tract disease.

## 1 Introduction

Allogeneic hematopoietic cell transplantation (alloHCT) is a well-established treatment for hematological malignancies, bone marrow failure syndromes, and inborn errors of immunity (1). Before alloHCT, patients received a preparative regimen known as conditioning (1). Generally, conditioning consists of two main components: myelodepletion and lymphodepletion, which typically involve chemotherapy and/or total body irradiation and immunosuppression (1, 2). Conditioning regimens are classified according to dose intensity into myeloablative, reduced-toxicity, reduced-intensity, and non-myeloablative regimens (1).

One of the most common complications occurring almost exclusively after alloHCT is graft-versus-host disease (GVHD) (1–3). The pathophysiology of GVHD is complex and multi-stage (1, 2). Based on clinical features, time of onset, and pathophysiology, GVHD can be classified into acute and chronic (3). The incidence of acute GVHD (aGVHD) varies among centers, ranging from approximately 40% to 80% (3). The most frequently affected organs are skin, gastrointestinal tract, and liver. Notably, gastrointestinal involvement affects up to 60% of patients, is linked to a worse prognosis, and poses significant diagnostic and therapeutic challenges (2, 3). The incidence of chronic GVHD (cGVHD) is approximately 50% among all patients and can affect various organs (1). Gastrointestinal GVHD (GI GVHD) is diagnosed based on clinical symptoms, endoscopic findings, and histopathological results (2).

According to the EBMT guidelines on GVHD prophylaxis, calcineurin inhibitors (cyclosporine A or tacrolimus), and antimetabolites (methotrexate or mycophenolate mofetil) are recommended as standard agents for GVHD prevention (4). The regimen of GVHD prophylaxis depends on the intensity of conditioning and the type of donor (for instance, cyclophosphamide post-transplant (PT-Cy) in haploidentical or unrelated mismatched donors). Corticosteroids remain the first-line therapy for patients diagnosed with GVHD (acute or chronic), whereas second-line therapy used in steroid-refractory or steroid-dependent cases typically involves the use of ruxolitinib, an inhibitor of Janus kinases (4).

Serum proteins such as REG3α are becoming important markers of GVHD risk and severity as well as predictors of treatment response. Despite the identification of several promising markers, none have yet been fully approved for routine clinical use, either for diagnosis or monitoring (2).

The review aims to provide a comprehensive overview on GVHD biomarkers with a particular focus on the intestinal manifestations of the disease as well as an overview of emerging therapeutic strategies.

## 2 Materials and methods

A literature search was conducted in the PubMed database. The following keywords were used as search terms: “GVHD,” “acute GVHD,” “gastrointestinal GVHD,” “GVHD biomarkers,” and “GVHD treatment”. The search was limited to studies involving human subjects, published in English from 1995 to March 2025. The snowball strategy was employed, which involved a manual review of references from articles available on the online database and previously published reviews to identify further relevant studies. The following inclusion criteria were applied: studies published in English and availability of full-text articles.

## 3 Pathophysiology of gastrointestinal GVHD

The pathophysiology of gastrointestinal GVHD remains incompletely understood. The development of acute GVHD (aGVHD) typically progresses through three sequential phases, namely: conditioning-mediated tissue damage, donor T-cell activation, and target cell apoptosis. During this process, numerous pro-inflammatory cytokines, including interleukin-1 (IL-1) and tumor necrosis factor (TNF), are produced and released. As a result, host tissues are targeted by cellular effectors, including natural killer (NK) cells and effector T lymphocytes, ultimately leading to intestinal epithelial damage (5).

Conditioning-induced damage to the gastrointestinal tract also promotes the systemic translocation of microbial products, including lipopolysaccharide (LPS) and other pathogen-associated molecular patterns (PAMPs). This activates antigen-presenting cells (APCs) and further enhances donor T-cell activation (5). In addition, conditioning eliminates group 2 innate lymphoid cells (ILC2s), which help maintain an anti-inflammatory environment in the gastrointestinal tract. In contrast, group 3 innate lymphoid cells (ILC3s), which secrete IL-22 to protect intestinal stem cells (ISCs) and stimulate the production of regenerating islet-derived protein 3 alpha (REG3α) by Paneth cells, remain resistant to conditioning (6).

GVHD develops when donor T cells recognize human leukocyte antigen (HLA) disparities on recipient tissues. Experimental models have demonstrated that host APCs are both essential and sufficient to activate donor T cells and initiate GVHD (5). Following allogeneic hematopoietic cell transplantation (alloHCT), activated donor T lymphocytes stimulate the release of alarmins, such as IL-33, and promote the release of soluble ST2, the decoy receptor for IL-33, from APCs (6). In the later phase of GVHD, donor T lymphocytes eliminate ILC3s, Paneth cells, and ISCs. This disruption fosters the overgrowth of potential pathogens, compromises the epithelial barrier, and triggers the release of REG3α (previously stored in mucus and Paneth cells) into the bloodstream (6). Finally, innate and adaptive immune cells amplify T-cell-mediated inflammation. Cytotoxic T lymphocytes and NK cells destroy target cells via Fas/Fas ligand (FasL) and perforin/granzyme pathways, while pro-inflammatory cytokines exacerbate tissue damage and contribute to multi-organ dysfunction (5).

## 4 Biomarkers

### 4.1 REG3α

REG3α is an antimicrobial peptide produced by Paneth cells that acts as a survival factor for intestinal stem cells (ISCs) and is critical for crypt regeneration (7). Acting downstream of IL-22, REG proteins help maintain the epithelial barrier integrity of the intestinal mucosa by binding bacterial peptidoglycans (8). REG3α is released into the bloodstream as a result of crypt cell damage caused by activated donor T lymphocytes, which typically occurs during the activation and effector phases of GVHD (6). Serum REG3α concentration, currently recognized as a key marker of gastrointestinal GVHD, has demonstrated a predictive value for both treatment response and non-relapse mortality (NRM) when measured at GVHD onset (7).

The first major study on biomarkers in GI GVHD was conducted by Ferrara et al. in a cohort of 871 patients (7). It was demonstrated that the concentration of REG3α in patients with GI GVHD was threefold higher than in other patients, including those with non-GVHD enteritis. In this study, in a group of 26 patients with clinical stage IV of GI GVHD at onset, 23 received full-intensity conditioning, and these patients exhibited a trend of higher REG3α concentrations compared with patients with stages I–III of GI GVHD. The prognostic value of plasma REG3α levels measured at the time of diagnosis of lower GI GVHD was also evaluated in 162 patients, revealing higher levels in patients who failed to respond to therapy after 4 weeks compared to those who achieved a complete or partial response. Additionally, NRM was approximately twice as high in patients with elevated REG3α concentrations, and this difference remained statistically significant after adjustment for known risk factors such as donor type, degree of HLA match, conditioning intensity, age, and baseline disease severity (*P* < 0.001) (7).

Zhao et al. reported an inverse correlation between plasma REG3α levels and Paneth cell counts in a cohort of 28 patients with GVHD (9). In patients with gastrointestinal GVHD, persistently high REG3α concentrations, frequently increasing by an order of magnitude after 1 week of systemic steroid therapy, were predictive of increased NRM. The authors also observed that IL-22 administration restores gastrointestinal epithelial integrity and alleviates GVHD, with REG3α and REG3γ acting as survival signals for ISCs and Paneth cells, preventing their apoptosis in *in vitro* and *in vivo* models (9).

In a study covering 954 alloHCT recipients, Harris et al. demonstrated that serum REG3α, hepatocyte growth factor (HGF), and cytokeratin fragment 18 were significantly elevated in patients with GI GVHD compared to those with non-GVHD diarrhea and asymptomatic controls (10). All three biomarkers measured at the onset of GI GVHD effectively predicted nonresponse to therapy at day 28, whereas REG3α and HGF also served as reliable prognostic markers of 1-year NRM in patients with lower GI GVHD (10). The combination of these biomarkers provided only a marginal improvement over REG3α alone (10).

Weber et al. conducted a study involving 587 patients treated with alloHCT and investigated REG3α serum concentrations on the day of alloHCT. The authors found that transplant-related mortality (TRM) was higher in the high-REG3α group due to GI GVHD. In contrast, REG3α concentration at preconditioning did not correlate with 1-year TRM (11). These findings suggest that early post-transplant REG3α serum concentrations may predict long-term outcomes in alloHCT recipients (11).

While the above-mentioned studies focused on the acute form of GVHD, DePriest et al. investigated the REG3α marker in chronic GI GVHD and demonstrated that high REG3α levels correlated with GI-cGVHD, but not with systemic markers, and were also associated with increased NRM (12). Collectively, these findings support the clinical utility of REG3α as a prognostic biomarker for severe GI GVHD and as a predictor of treatment nonresponse. Nonetheless, optimal time points and cutoff values remain to be determined.

### 4.2 ST2

Suppressor of tumorigenesis 2 (ST2), a receptor for IL-33, has three known isoforms, namely: membrane-bound, soluble (sST2), and a variant form (13). IL-33, a member of the IL-1 superfamily, is a multifunctional protein that plays immune-modulatory roles both as an alarmin and a pleiotropic cytokine. It has been demonstrated that IL-33 plays a critical role in the maintenance and regulation of the ST2+ Treg population and promotes the chemotaxis of dendritic cells and neutrophil granulocytes. The soluble form acts as a decoy receptor, blocking IL-33 signaling and inhibiting lipopolysaccharide (LPS)-induced cytokine production. Notably, it is this soluble form that is currently under investigation as a potential biomarker for GVHD (13). Substantial amounts of sST2 are released into the bloodstream as a result of the intensified inflammatory cascade and elevated secretion of IL-33 by immune cells. However, sST2 may also be released due to tissue injury caused by conditioning. Reichenbach et al. assessed the activity of the IL-33/ST2 axis in patients with GVHD. They showed that the interaction between IL-33 and sST2 on T cells enhances IFN-γ production, induces IL-18R expression, and promotes cell proliferation, contributing to the GVHD progression. An increased production of endogenous IL-33 was observed in the gastrointestinal (GI) tract of patients with GVHD and in non-hematopoietic cells within the GI tract of GVHD mice (13).

One of the first studies investigating ST2 was conducted by Vander et al. (14). They demonstrated that higher serum sST2 levels at therapy initiation were associated with a 2.3-fold increased risk of treatment-resistant GVHD compared to lower serum sST2. In univariate analysis, age, HLA match, GVHD grade at therapy initiation, and initial treatment for GVHD were associated with 6-month post-therapy non-relapse mortality. After adjusting for demographic and clinical variables, higher sST2 values remained a significant predictor of increased risk of death compared to lower values (14).

The impact of sST2 expression prior to transplantation on NRM and GVHD occurrence was examined by Gjærde et al. in 374 patients (15). It was found that patients who had 1-year NRM exhibited significantly higher sST2 levels on day +7 post-transplantation and day +14, and their pre-transplantation sST2 concentrations were also higher, but the difference was not significant. Notably, the rise in sST2 levels from pre-transplantation to day +7 predicted both 1-year NRM and the development of aGVHD, whereas the change in levels by day +14 served as a prognostic indicator of 1-year NRM. Additionally, sST2 levels at day +7 were associated with both 1-year all-cause mortality and aGVHD, while pre-transplantation sST2 levels were associated only with 1-year all-cause mortality, but not with aGVHD or relapse (15).

In a study involving 113 patients, Ponce et al. examined sST2 concentrations on day 28 post-transplant (16). The median sST2 level was 33.9 ng/mL and was used as a threshold to distinguish between patients with high and low sST2 concentrations. Patients with high sST2 levels had a cumulative incidence of 66% aGVHD grades II–IV at day 180, compared with 52% in those with low sST2 levels (*p* = 0.048). Specifically for GI involvement, the incidence of grades II–IV was 26% in the high-sST2 group versus 16% in the low-sST2 group (*p* = 0.245). Additionally, a high sST2 concentration on day 28 was predictive of increased TRM at day 180, with a rate of 23% compared to 5% in patients with low serum sST2 (16).

Matsumura et al. evaluated ST2 and IL-33 concentrations in plasma before conditioning, on the day of alloHCT, and on days 14, 21, and 28 following alloHCT in a cohort of 32 patients (17). ST2 concentrations varied by conditioning intensity and were higher in patients receiving reduced-intensity conditioning (RIC). On day 14, higher ST2 levels correlated with an increased risk of aGVHD, although these differences were not statistically significant. The incidence of aGVHD grades II–IV was significantly higher in the group with elevated ST2 levels (56.7% vs. 16.5%, *p* < 0.01). It was also demonstrated that in the group with higher ST2, GI aGVHD was significantly more frequent (50% vs. 15%, *p* = 0.03), but there was no association with the cutaneous form of the disease. Patients with high sST2 concentration on day 14 had a significantly higher 1-year cumulative incidence of NRM compared to those with low sST2 (33% vs. 0%, *p* < 0.01) (17).

Overall, high plasma sST2 concentrations measured early after transplantation consistently predict a poor response to GVHD therapy and increased non-relapse mortality, independent of GVHD clinical grade. This provides superior risk stratification compared with conventional clinical parameters and supports the utility of sST2 as a prognostic biomarker. However, standardized timing and threshold values for measurement have yet to be established, and sST2 levels may be influenced by post-transplant complications or comorbidities.

### 4.3 TIM-3

T cell immunoglobulin and mucin domain 3 (TIM-3) is a type I transmembrane receptor that suppresses T helper 1 (TH1) cell activation upon binding to its ligand, galectin-9 (Gal-9), and plays a key regulatory role in autoimmune disorders, chronic infections, anti-tumor immunity, and transplantation (18). The release of sTIM-3 into the bloodstream mainly results from T-cell activation. During acute GVHD, donor T cells rapidly upregulate TIM-3, while non-hematopoietic cells increase the expression of galectin-9, although its exact role in GVHD pathogenesis remains incompletely understood (19). Hansen et al. conducted a study on 127 patients following alloHCT, measuring soluble TIM-3 (sTIM-3) weekly from days 7 to 35, on days 56 and 80, and at the onset of aGVHD (18). Patients with severe mid-gut GVHD had significantly higher plasma TIM-3 concentrations than those with upper-gut GVHD (*p* = 0.005), patients without GVHD (*p* = 0.002), and healthy controls (*p* < 0.0001). Additionally, the surface expression of TIM-3 was elevated on CD8+ T cells in patients with grades II–IV acute GVHD (*p* = 0.01) (18).

Yegin et al. conducted a prospective study in a group of 177 patients treated with alloHCT, in whom TIM-3 was measured before transplantation. A positive correlation was observed between baseline TIM-3 and the severity of GVHD (*p* = 0.013) (20). Veenstra et al. explored the TIM-3/galectin-9 pathway blockade, demonstrating that the inhibition of TIM-3/Gal-9 in the absence of donor T-regulatory cells (Tregs) resulted in GVHD suppression, suggesting that this pathway could serve as a potential therapeutic target (19).

### 4.4 TNFR1

Tumor necrosis factor receptor 1 (TNFR1) is mainly released by activated monocytes, macrophages, and T cells (21). TNFR1 is ubiquitously expressed and initiates pro-inflammatory signaling pathways. TNF also signals through a second receptor, TNFR2, which, in contrast to TNFR1, is restricted to specific immune cell subsets and primarily mediates immunoregulation and cell proliferation (22). The concentration of soluble TNFR1 (sTNFR1) strongly correlates with TNF but is more stable during long-term storage; therefore, it is considered a candidate biomarker for GVHD (21). Both TNF and sTNFR1 are produced and released by macrophages and activated T lymphocytes in response to tissue injury caused by conditioning regimens. They are also involved in the subsequent phases of GVHD, including donor T-cell activation (23).

Kitko et al. studied sTNFR1 expression in a group of 82 pediatric patients on day 7 post-alloHCT and showed that elevated sTNFR1 was associated with an increased severity of GVHD (*p* = 0.02). Moreover, a baseline sTNFR1 ratio greater than 2.5 was linked to a poorer 1-year overall survival (51% vs. 74%, *p* = 0.04) and a higher incidence of TRM (21).

Willems et al. studied TNFR1 before conditioning and on day 7 post-alloHCT and demonstrated that a high ratio of day 7 TNFR1 to pre-conditioning sTNFR1 was associated with grades II–IV aGVHD (*p* = 0.01) and grades III–IV aGVHD (*p* = 0.007) (23). A similar study was conducted by Choi et al. in a group of 438 patients, in which sTNFR1 concentration in plasma was measured both before transplantation and at day 7 post-transplant (24). The study showed that an increase of TNFR1 greater than or equal to 2.5-fold over baseline correlated with a higher risk of grades II–IV aGVHD (58% vs. 32%, *p* < 0.001) and was associated with more than double likelihood of death from 1-year TRM (39% vs. 17%, *p* < 0.001) (24).

Pedraza et al. measured the concentrations of VCAM-1, sTNFR1, and VWF: Ag as markers of endothelial damage before conditioning immediately before transplantation (day 0) and on days 3, 7, 14, and 21 after alloHCT in 321 patients while also calculating the endothelial activation and stress index (EASIX) (25). An association was found between high sTNFR1 and log-2 EASIX score on day 7 after transplantation with the occurrence of aGVHD (*p* = 0.002 and *p* = 0.013, respectively), higher risk of grade II-IV aGVHD, and a significantly higher risk of NRM (25).

A similar study was conducted by Mir et al. in a group of 31 patients with aGVHD in whom the VWF, ADAMTS-13, and sTNFR1 were measured on days –1 (preconditioning), 0, and 7 and compared with a group of alloHCT patients who did not develop aGVHD (26). The high concentration of VWF and sTNFR1 on day 7 after alloHCT correlated with the development of aGVHD (*p* = 0.0002 and *p* < 0.01, respectively) (26).

Overall, sTNFR1 emerges as a prognostic biomarker for the risk of severe GVHD and TRM.

### 4.5 IL-6

IL-6 exerts pleiotropic effects, including the maturation and activation of B cells, T cells, and macrophages (27). Systemic IL-6 levels become dysregulated after alloHCT. After transplantation, IL-6 is primarily produced by recipient cells, particularly by dendritic cells (28).

In a study involving 166 patients, Greco et al. examined IL-6 as a biomarker for aGVHD and studied survival following alloHCT with the post-transplant use of cyclophosphamide (29). IL-6 was measured before conditioning and 7 days following alloHCT. Patients with IL-6 above 16.5 pg/mL on day 7 after transplantation were more likely to develop grades II–IV and III–IV aGVHD and particularly grades II–IV gastrointestinal involvement. High post-transplant IL-6 levels were also associated with a higher risk of steroid-refractory aGVHD. Notably, 94% of patients with steroid-refractory GVHD (SR-GVHD) had elevated serum IL-6. Pre-transplant IL-6 >2.5 pg/mL was associated with the development of grades II–IV aGVHD. Additionally, patients with elevated IL-6 levels had increased TRM and relapse rates (29).

### 4.6 GLP-2

Glucagon-like-peptide-2 (GLP-2) is an enteroendocrine hormone produced by intestinal L cells, which exerts a protective and regenerative role in the gastrointestinal mucosa through direct and indirect effects on responsive cells (30, 31). Studies have shown that GLP-2 promotes crypt cell proliferation, enhances mesenteric blood flow, and stimulates mucosal growth. Its other functions include improving nutrient absorption, elongating villi, improving barrier function, and reducing gut permeability and motility as well as suppressing epithelial cell apoptosis and inflammation (30, 32). It also modulates intestinal epithelial cells, such as intestinal stem cells (ISCs), Paneth cells (PCs), and goblet cells, helping to preserve the integrity of the GI tract (33). Cytotoxic conditioning before alloHCT damages mainly intestinal L cells. Preclinical studies have shown that an extensive loss of intestinal stem cells and Paneth cells is linked to aGVHD and results in the disruption of GI homeostasis (30, 34). Norona et al. observed a decline in GLP-2+ L cell levels in mice that developed aGVHD as well as a concurrent decrease in GLP-2R expression (30). In GI GVHD, the concentration of GLP-2 in intestinal tissues is decreased due to damage to the mucosal lining and the loss of enteroendocrine L-cells caused by activated donor T lymphocytes. As a result, GLP-2 is released into the systemic circulation, and the remaining L-cells increase the GLP-2 production as a compensatory response, leading to elevated GLP-2 levels in the bloodstream. A study conducted by Norona et al. in a cohort of patients with acute gastrointestinal GVHD demonstrated a higher incidence of SR-GVHD in patients with fewer than one L-cell per crypt and lower NRM in patients with one or more L-cells per crypt (30).

Elevated circulating GLP-2 at the time of GVHD diagnosis was associated with increased risks of SR-GVHD and NRM. Importantly, high GLP-2 levels also predicted poor outcomes in patients without gastrointestinal symptoms (30). Thus, GLP-2 may serve as both a prognostic marker for severe, steroid-refractory GVHD and a predictive marker of treatment response. However, larger prospective studies are required to validate and standardize its clinical utility.

### 4.7 Amphiregulin

Amphiregulin (AREG) is a ligand of the epidermal growth factor receptor (EGFR), primarily produced by type 2 innate lymphoid cells (ILC2s) in an IL-33-dependent manner. It is also secreted by other cell types, including regulatory T cells (Tregs), fibroblasts, keratinocytes, dendritic cells, CD4^+^ T cells, and granulocytes (35). AREG plays a pivotal role in type 2 immune responses, particularly by supporting epithelial repair and regeneration following tissue injury (35). Circulating AREG increases both following tissue damage from conditioning regimens and, later, as a result of immune activation in the course of GVHD, particularly by ILC2s and Tregs (36).

In a study of 48 patients with GVHD, Amin et al. observed that individuals with high gastrointestinal AREG expression but low circulating AREG levels had the highest 1-year survival rate (71%). In contrast, patients with high serum AREG concentrations demonstrated markedly reduced survival rates (<30%), irrespective of local tissue expression (36). Furthermore, circulating AREG levels ≥ 33 pg/mL were associated with lower response rates to steroid therapy and increased mortality (36). Holtan et al. analyzed serum/plasma AREG concentrations at the onset of acute GVHD (aGVHD) symptoms in a cohort of 251 patients (35). Among patients classified as standard risk according to the Minnesota criteria, those with AREG ≥33 pg/mL had significantly lower rates of complete or partial response (CR/PR) by day 28 (*p* = 0.02), increased non-relapse mortality (NRM) (*p* < 0.01), and inferior overall survival (OS) (*p* < 0.001). Additionally, the AREG levels at GVHD onset were approximately twofold higher in patients with stages II–IV lower GI tract involvement than in those without such involvement (*p* < 0.01) (35).

In a separate analysis involving 83 patients with late-onset aGVHD, Holtan et al. found elevated plasma AREG concentrations. However, the AREG levels alone were not significantly associated with OS (*p* = 0.11) or NRM. Notably, an elevated AREG-to-EGF ratio was associated with increased NRM and inferior OS (37). Furthermore, in a phase II trial involving 22 patients with high-risk aGVHD (Minnesota classification), Holtan et al. assessed the addition of urinary-derived human chorionic gonadotropin and epidermal growth factor (hCG/EGF) to methylprednisolone therapy. Persistently elevated plasma AREG was observed in nonresponders, correlating with AREG expression on peripheral blood T cells and plasmablasts (38). Overall, amphiregulin has emerged as a prognostic biomarker for severe and treatment-refractory GVHD.

### 4.8 Soluble IL-2 receptor

Activated donor T lymphocytes upregulate the expression of the interleukin-2 receptor alpha subunit (CD25). The receptor is subsequently released into the bloodstream in a soluble form (sIL-2Rα). Elevated sIL-2Rα concentrations are considered an early indicator of T-cell activation and have been linked to both the development and severity of GVHD (39).

In a study of 43 patients after alloHCT, Mathias et al. measured sIL-2Rα concentrations weekly for 4 weeks and demonstrated a significant association between elevated levels and a clinical diagnosis of aGVHD (*p* < 0.001) (40). Similarly, Nakamura et al., in a cohort of 18 patients, reported that the sIL-2Rα levels measured before and after alloHCT were strongly associated with GVHD severity (*p* < 0.001) (41). Foley et al. monitored the weekly sIL-2Rα levels in 36 patients. They observed no significant differences between matched related and unrelated donors. However, patients who developed aGVHD (53%) had significantly elevated sIL-2Rα levels in weeks 2 and 3 (*p* = 0.02 and *p* = 0.04, respectively). Increased concentrations were also detected in patients with sepsis (week 4, *p* = 0.02) and veno-occlusive disease (week 2, *p* = 0.03) (39). Similarly, Miyamoto et al. reported a correlation between the peak serum concentration of sIL-2Rα and aGVHD severity in a cohort of 30 post-alloHCT patients (42). In a study of 27 patients, Kobayashi et al. observed significant increases in sIL-2Rα at both engraftment and the onset of aGVHD (43). Collectively, these findings indicate that sIL-2Rα is a consistent biomarker of T-cell activation and may serve as an early indicator of both the onset and severity of GVHD.

### 4.9 Novel potential biomarkers

McCarthy et al. investigated Gal-3, LAG-3, PD-1, IL-6, TIM-3, TNFR1, REG3α, and ST2 concentrations on days 7, 14, and 21 after alloHCT. On days +7 and +14, the Gal-3 concentrations were significantly higher in patients who developed grades II–IV aGVHD compared with those who developed grades 0–I disease (44).

In a cohort of 170 pediatric patients, Berger et al. measured TNFR1, IL-2Rα, hepatocyte growth factor (HGF), monocyte chemoattractant protein-2 (MCP-2), IL-8, and IL-12p70 on days −1, +1, +7, +14, +21, +28, and +60 (45). The elevated IL-2Rα and HGF levels on days +14 and +21 were strongly associated with an increased risk of aGVHD. On day +14, grades II–IV occurred in 60% vs. 28% of patients (*p* = 0.007) and grades III–IV in 40% vs. 15% (*p* = 0.001). By contrast, the TNFR1, CCL8, IL-8, and IL-12p70 levels showed no significant correlation with aGVHD incidence (45).

### 4.10 Combined scores for the prediction of aGVHD courses

To enhance predictive accuracy, several groups have developed algorithms combining multiple biomarkers. Levine et al. developed and validated an algorithm based on plasma biomarker concentrations from 492 patients to predict the probability of 6-month NRM at aGVHD onset (46). This biomarker-based algorithm, termed the Ann Arbor score, improved the prediction of treatment response and NRM compared with clinical assessment alone (46).

The Mount Sinai Acute GVHD International Consortium (MAGIC) evaluated 507 patients from 17 centers (47). This study assessed an algorithm using REG3α and sST2 to predict NRM and steroid resistance in aGVHD. Marker concentrations were measured on day 7 after the initiation of steroid treatment. Measurements of sST2 and REG3α at the time of clinical response allowed the identification of slow responders (47).

In a multicenter study involving 1,287 patients after alloHCT, Hartwell et al. measured sST2, REG3α, sTNFR1, and IL-2Rα levels 7 days post-alloHCT (48). Based on sST2 and REG3α, an algorithm was created to classify patients into high-risk and low-risk groups. In the high-risk group, GVHD-related mortality was 18% compared to 4% in the low-risk group (*p* < 0.001). Severe GI GVHD also occurred more frequently in the high-risk group (17% vs. 8%, *p* < 0.001). This model also identified patients with a cumulative incidence of 6-month NRM of 28% in the high-risk group compared to 7% in the low-risk group (*p* < 0.001) (48).

Other biomarker models have also been explored. McDonald et al. demonstrated that plasma TIM-3, sST2, and sTNFR1 levels could predict treatment failure and NRM in 165 patients with aGVHD after 14 days of steroid therapy (49). However, these models lacked sufficient positive predictive value to identify high-risk GVHD cohorts for investigational trials (49).

Leotta et al. measured the plasma concentrations of sIL-2Rα, TIM-3, ST2, intercellular adhesion molecule (sICAM-1), IFN-γ, and IL-6 on day 18 after alloHCT in a cohort of 95 patients (50). The incidence of aGVHD was higher among patients with both TIM-3 and sIL-2Rα concentrations above the threshold (54% vs. 36%, respectively), although this difference was not statistically significant (50). GI GVHD occurred in 33% of patients with both TIM-3 and sIL-2Rα above the threshold, in 8.5% with one marker elevated, and in 7.1% with both markers below the threshold (*p* = 0.007). When analyzed as continuous variables, the plasma concentrations of sIL-2Rα and TIM-3 demonstrated a predictive value for overall survival (sIL-2Rα, *p* = 0.002; TIM-3, *p* = 0.0007), while TRM was predicted by sIL-2Rα (*p* = 0.0005), IFN-γ (*p* = 0.01), and IL-6 (*p* = 0.0001) (50).

Etra et al. compared REG3α, sST2, and amphiregulin markers as biomarkers of disease severity at the time of GI GVHD diagnosis (51). Both algorithms proved highly effective in predicting the 6- and 12-month NRM, respectively. Although other GI GVHD marker combinations performed reliably, adding AREG to sST2 and REG3α did not improve the predictive performance (51). Robin et al. evaluated the weekly plasma levels of elafin, HGF, IL2-Rα, IL8, REG3α, sST2, and sTNFRI until week 7 after alloHCT and at aGVHD onset in 204 patients (52). However, these biomarkers provided only marginal improvements in predicting overall survival and NRM.

Balakrishnan et al. measured sST2, REG3α, VCAM1, ICAM1, and TIM-3 before conditioning, after conditioning, and on days 14 and 28 following alloHCT in 56 patients and on day 14 after alloHCT in an additional 154 patients. The sST2 concentration on day 28 and ICAM1 on day 14 showed a prognostic significance for aGVHD, particularly in GI GVHD (53). Of the algorithms described above, only the MAGIC algorithm is currently implemented in clinical practice, whereas the others still require validation in larger standardized studies.

## 5 Selected emerging therapeutic strategies for GVHD

### 5.1 GLP-2 analogues

**Figure 1:**
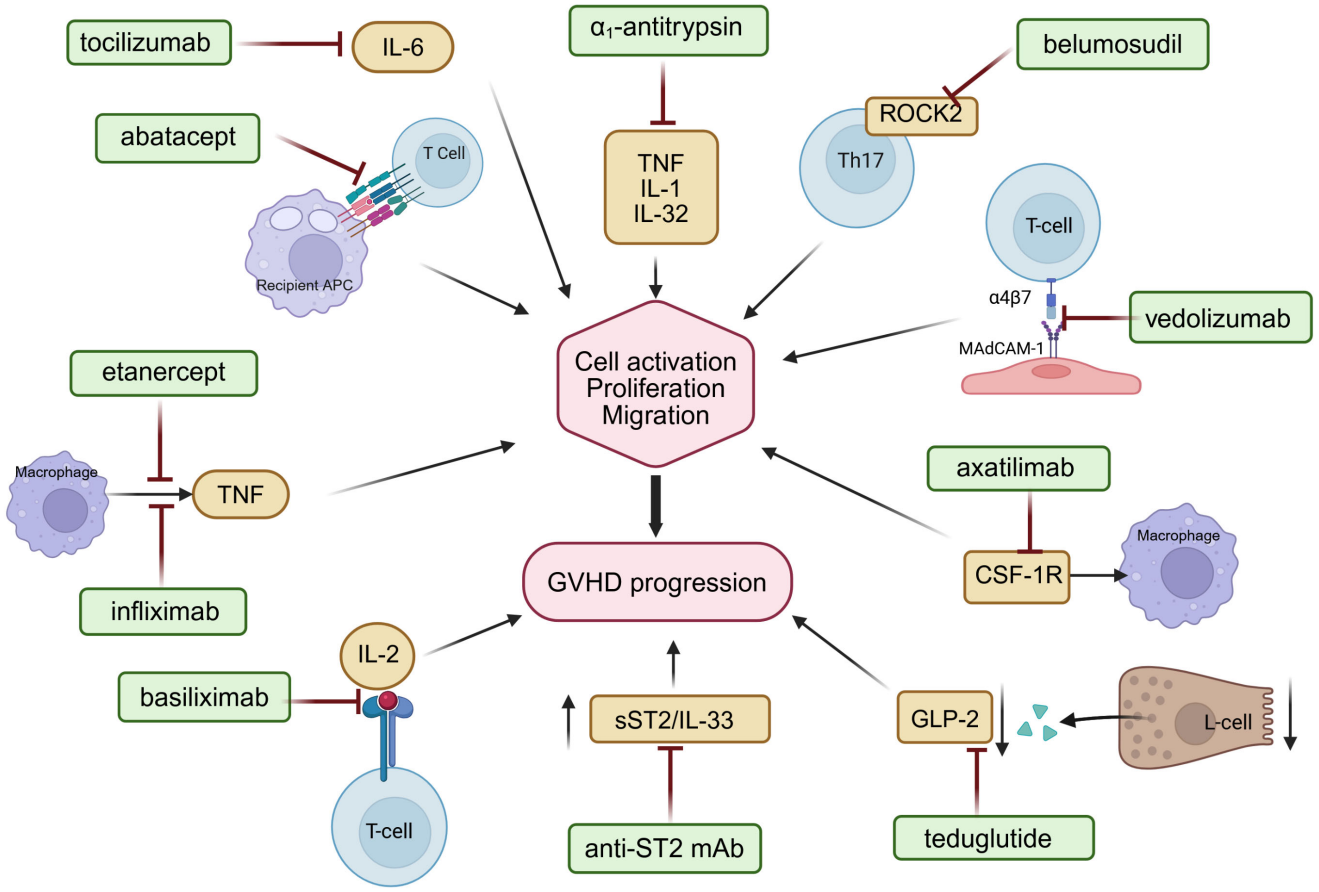
Mechanisms of action of selected immunomodulatory agents targeting key pathogenic pathways in acute and chronic GVHD, including costimulatory blockade, cytokine signaling inhibition, and T-cell trafficking modulation. Interleukin-6 (IL-6), interleukin-1 (IL-1), interleukin-32 (IL-32), interleukin-2 (IL-2), tumor necrosis factor (TNF), Rho−associated coiled−coil containing protein kinase 2 (ROCK2), colony-stimulating factor-1 receptor (CSF-1R), glucagon-like peptide-2 (GLP-2), soluble suppression of tumorigenicity 2/interleukin 33 pathway (sST2/Il-33). Figure created with BioRender.com.

Teduglutide, a GLP-2 analogue, exhibits improved stability and an extended half-life compared with native GLP-2. It has been investigated in phase 3 clinical trials in patients with short bowel syndrome (30). In a murine transplantation model, Norona et al. investigated the prophylactic potential of teduglutide (30). Teduglutide prophylaxis reduced GVHD-related mortality and limited organ involvement in histopathological assessment. Treatment with teduglutide from day −3 to +10 increased the intestinal GLP-2R expression relative to controls, suggesting the mitigation of acute SR-GVHD severity *in vivo*. Treatment also enhanced the mRNA expression of Paneth cell markers, such as lysozyme, Reg3γ, and Defα4 (30). These findings indicate that Paneth cell loss during GVHD can be partially reversed by exogenous GLP-2, leading to increased Paneth cell numbers and higher levels of antimicrobial factors, including lysozyme and α-defensins (30) (**Figure 1**). The impact of teduglutide on intestinal microbiome diversity in aGVHD mice was also evaluated. Teduglutide treatment also modified the microbiome, notably increasing the relative abundance of unclassified Bacteroidales. Given that intestinal interferon signaling reflects bacterial translocation, the teduglutide-associated downregulation of interferon-related genes suggests reduced translocation (30). Moreover, GVHD mice exhibited lower keratinocyte growth factor (KGF) concentrations, whereas those treated with teduglutide showed elevated KGF levels. Several ISC markers were also upregulated in the teduglutide-treated group compared with the controls (30).

Ramos et al. described three cases of off-label teduglutide use in patients with steroid-resistant GI GVHD (54). Teduglutide administration markedly alleviated disease symptoms and promoted endoscopic remission. As a potent non-immunosuppressive agent, teduglutide may represent an early adjunctive therapy for acute GI GVHD, especially in high-risk patients identified by biomarkers or risk algorithms (54).

Apraglutide is a next-generation synthetic GLP-2 analogue administered once weekly (34, 55). In preclinical models, apraglutide reduced villous atrophy and colon shortening while improving weight gain and survival after total body irradiation and alloHCT-induced aGVHD (34).

### 5.2 Blockade of soluble ST2

Zhang et al. demonstrated that soluble ST2 (sST2) concentrations were significantly elevated in mice after alloHCT, even prior to the clinical onset of GVHD (56). A daily administration of anti-ST2 monoclonal antibody (mAb) from days -1 to +9 after alloHCT significantly attenuated the GVHD severity and improved the survival (56). The short-term blockade of sST2 achieved by administering anti-ST2 mAb 1 day before and 1 day after alloHCT proved effective as prophylaxis against GVHD. Similarly, in a human-to-mouse xenogeneic GVHD model, sST2 blockade effectively alleviated the symptoms and improved the survival. Organ-specific analysis revealed the highest sST2 levels in the intestine, primarily derived from endothelial cells in the GI tract damaged during pre-transplant conditioning (56). Consistently, Yuan et al. reported that mice treated with an ST2 inhibitor displayed significantly milder GVHD manifestations and improved overall survival (57).

### 5.3 Tocilizumab

Tocilizumab (TCZ) is a humanized monoclonal antibody targeting the IL-6 receptor. It is EMA-approved for the treatment of severe active rheumatoid arthritis in adults and juvenile idiopathic polyarthritis. Furthermore, TCZ has a well-established role in the treatment of cytokine release syndrome (CRS) after CAR-T cell therapy (1).

Chen et al. showed significantly elevated IL-6 concentrations in mice after bone marrow transplantation compared with the controls. Additionally, a histopathological analysis revealed fewer pathological changes in the colon, liver, and lungs of mice with IL-6 blockade (58).Clinical data on tocilizumab in SR-GVHD remain limited. Drobyski et al. reported eight patients with SR-GVHD who received tocilizumab at 8 mg/kg once weekly every 3 to 4 weeks. Responses were observed in the four patients with aGVHD (two complete and two partial remissions) and one with cGVHD, for an overall response rate of 67% (59). Roddy et al. reported on nine patients with GI GVHD who received tocilizumab as a second- or third-line agent. In this case series, the overall response rate (ORR) was 44%, with two partial and two complete responses (CR); however, six patients died from aGVHD and related complications (60).

Kennedy et al. assessed tocilizumab as an adjunct to GVHD prophylaxis in a phase 1/2 clinical trial involving 48 patients (61). By day 100 after alloHCT, grades III/IV aGVHD occurred in 4% of tocilizumab-treated patients and grades II–III in 12%. The relapse rate was 27%. During a median follow-up of 497 days, 51% of the patients developed cGVHD (61).

A randomized, double-blind phase 3 trial involving 145 patients assessed TCZ efficacy in preventing grades II–IV aGVHD (62). At day 100, grades II–IV GVHD occurred in 36% of controls and 27% of TCZ-treated patients (*p* = 0.23). In the unrelated donor (URD) subgroup, the incidence was 45% versus 32% (*p* = 0.16). Notably, these differences did not reach statistical significance. No organ-specific protection against grades II–IV aGVHD was evident when comparing the placebo and TCZ groups. However, the incidence of stages III–IV skin events (11 vs. 8), stages I–IV gastrointestinal events (13 vs. 9), and stages I–IV liver events (eight vs. three) was lower among TCZ recipients (62).

Additional retrospective studies also support potential benefit. Kattner et al. carried out a retrospective analysis of 11 patients with steroid-refractory cGVHD treated with TCZ, reporting a best overall response rate of 70% and a median time to response of 3 months (63). Ganetsky et al. conducted a retrospective study of 16 patients with lower gastrointestinal involvement in steroid-refractory aGVHD. Of 16 patients, 10 achieved CR after a median of 11 days from TCZ initiation; four responded after a single dose, while six required multiple doses to attain CR (64).

### 5.4 Human interleukin-22 dimer

Interleukin-22 (IL-22) is a tissue-protective cytokine that promotes mucosal healing and strengthens the intestinal barrier by acting directly on epithelial cells. In experimental models, aGVHD reduces the number of intestinal stem cell and depletes IL-22-producing cells, leading to more extensive epithelial damage and increased GVHD-related mortality after bone marrow transplantation (65).

Ponce et al. conducted a multicenter, single-arm phase 2 study in 27 patients to evaluate a recombinant human interleukin-22 dimer (F-652) plus systemic corticosteroids for newly diagnosed lower gastrointestinal aGVHD. Among 27 evaluable patients, 19 (70%) achieved a response in the lower GI tract by day 28, including 13 CRs, three very good partial responses, and three PRs. Interestingly, the responders showed a distinct fecal microbiota profile characterized by the expansion of commensal anaerobes and increased overall α-diversity, indicating an improvement in GVHD-associated dysbiosis (65).

### 5.5 TNF antagonists

Etanercept consists of two recombinant human TNFR (p75) monomers attached to the Fc portion of human IgG1, which binds to TNF and neutralizes its activity (66). In a prospective study, Choi et al. evaluated etanercept (25 mg twice weekly from the start of conditioning to day +56) compared with standard prophylaxis in 100 patients. On day +7 post-transplant, TNFR1 expression was unchanged after TBI-based conditioning but was 40% lower after non-TBI conditioning. This reduction correlated with less severe grades III–IV aGVHD (14%), reduced 1-year NRM (16%), and improved 1-year survival (69%) (66).

Levine et al. compared methylprednisolone alone versus methylprednisolone plus etanercept in 61 patients with aGVHD (67). The patients treated with etanercept were significantly more likely to achieve a CR than those treated with steroids alone (69% vs. 33%; *p* < 0.001). Additionally, plasma TNFR1 was elevated at GVHD onset and declined significantly only in CR patients (67). Gatza et al. demonstrated that adding etanercept to topical corticosteroids lowered the progression to grades II–IV aGVHD (29% vs. 43%) and reduced the 1-year grades II–IV (41% vs. 61%) and grades III–IV (3% vs. 18%) incidence versus the controls (68).

Additional reports showed variable efficacy. Park et al. reported that etanercept exerted a suppressive effect on aGVHD (69). De Jong et al. conducted a retrospective study of 15 patients with steroid-resistant grade III GI GVHD treated with etanercept. The ORR was 53%, with median overall survival of 99 days in responders versus 17 days in non-responders (*p* < 0.01) (70). Van Groningen et al. studied 21 patients with severe steroid-refractory aGVHD treated with combined inolimomab (anti-IL-2Rα) and etanercept. The combination therapy did not improve the clinical outcomes (71).

In a phase III clinical trial conducted by Couriel et al. involving 63 patients, the study group received methylprednisolone (MP) plus infliximab, while the control group received methylprednisolone alone. The response rates at days 7 and 28 for infliximab + MP vs. MP were 52% vs. 78% (*p* = 0.03) and 62% vs. 58% (*p* = 0.7), indicating no meaningful clinical benefit (72).

### 5.6 Vedolizumab

Vedolizumab is a humanized monoclonal antibody that selectively binds the α4β7 integrin on leukocytes. This blocks interaction with mucosal addressin cell adhesion molecule-1 (MAdCAM-1) expressed on gastrointestinal endothelial cells. By preventing the migration of α4β7-expressing T lymphocytes into the intestinal mucosa, vedolizumab helps attenuate gastrointestinal inflammation, making it a potential therapeutic option for gastrointestinal manifestations of GVHD (73).

Mehta et al. evaluated vedolizumab as a third-line therapy in 20 patients with grades III–IV gastrointestinal SR-GVHD who had previously failed both corticosteroids and ruxolitinib. The ORRs at days 14, 28, and 56 were 45%, 35%, and 25%, respectively (74). Notably, the responses were more pronounced in patients with prior ruxolitinib failure. However, 15 patients died during follow-up (74). Danylesko et al. reported an ORR of 79% (28% CR, 52% PR) in 29 patients with intestinal SR-GVHD treated with vedolizumab; it was used as second-line therapy in 45% and third-line therapy or later in 55% (75).

Fløisand et al. retrospectively analyzed 29 patients with gastrointestinal SR-GVHD and observed an ORR of 64% at 6 to 10 weeks after vedolizumab initiation. The 6-month overall survival rate was 54% (76). In a small retrospective cohort (*n* = 7), Zu et al. reported CR and PR rates of 57.1% and 42.9%, respectively; no patient developed cGVHD during follow-up (77).

Vedolizumab has also been explored for the prevention of gastrointestinal aGVHD. Goto et al. conducted a randomized study involving 333 patients undergoing alloHCT (78). Lower-GI aGVHD-free survival by day +180 was 94% with vedolizumab vs. 81% with placebo in Japanese patients (*p* = 0.2) and 84% vs. 70% in non-Japanese patients (*p* = 0.002) (78). In a similar randomized controlled trial (*n* = 343), Chen et al. showed a higher lower-GI aGVHD–free survival by day +180 with vedolizumab vs. placebo (85.5% vs. 70.9%; *p* < 0.001) (73).

Collectively, these data support vedolizumab as a therapeutic and prophylactic option in GI GVHD, particularly in steroid-refractory disease and for the prevention of lower-GI involvement after transplantation.

### 5.7 α_1_-Antitrypsin

α1-Antitrypsin (AAT) is a serine protease inhibitor produced in the liver. It protects tissues from proteolysis and exerts anti-inflammatory, anti-apoptotic, and immunomodulatory effects. In a phase II trial by Magenau et al., 40 patients with SR-GVHD received AAT twice weekly for 4 weeks (79). By day 28, the ORR was 65%, with a 35% complete response rate, demonstrating efficacy across all aGVHD target organs (79).

Gergoudis et al. conducted a multicenter clinical trial in 30 high-risk patients, identified by elevated REG3α and ST2 levels. Treatment with AAT was well tolerated and associated with minimal toxicity, but it did not significantly reduce the incidence of SR-GVHD compared with the controls (20% vs. 14%, *p* = 0.56) (80).

In a phase I/II open-label, single-center study, Marcondes et al. treated 12 patients with AAT as salvage therapy for SR-GVHD (81). The GVHD symptoms improved in eight of 12 patients, with four patients achieving a complete response (81). Giannoni et al. retrospectively analyzed 16 patients with gastrointestinal SR-GVHD treated with AAT (82). The ORR was 44%, including 27% CR, with gastrointestinal responses in 61% of patients (82).

### 5.8 Basiliximab

Basiliximab is a chimeric monoclonal antibody that binds selectively to the α-chain (CD25) of the IL-2 receptor on activated T cells. By blocking IL-2 binding, it inhibits T-cell activation and proliferation, which are the key mechanisms responsible for immune-mediated tissue injury such as GVHD (83).

Liu et al. evaluated basiliximab in 230 patients with SR-GVHD. The drug was administered on days 1, 3, and 8 and then weekly until aGVHD improved to below grade II or no response was observed after four doses. The cumulative ORRs at days 14, 28, and 56 were significantly higher in the basiliximab group compared to the controls: 41.4% vs. 23.1% (*p* = 0.023), 70.2% vs. 43.6% (*p* = 0.002), and 80.1% vs. 66.7% (*p* = 0.013), respectively (83).

Tan et al. conducted a prospective study in 65 patients with severe SR-GVHD treated with basiliximab plus etanercept. This regimen achieved higher CR rates in visceral aGVHD and significantly improved the 2-year OS compared with conventional salvage therapy (54.7% vs. 14.8%, *p* < 0.001) (84). Schmidt-Hieber et al. performed a phase II prospective study in 23 SR-GVHD patients treated with basiliximab. The primary ORR was 82.5%, including a CR in 17.5% of the patients and a PR in 65% (85). Chakupurakal et al. studied 14 SR-GVHD patients treated with basiliximab. The ORR was 92% (13/14), with 50% CR (7/14). However, GVHD recurred in 54% (7/13) of the initial responders (86).

In a study of 53 SR-GVHD patients, Wang et al. reported 46 responses to basiliximab, including 37 complete remissions. The median time to response was 6 days from treatment initiation (87). Massenkeil et al. assessed the efficacy of basiliximab in 17 patients with SR-GVHD, reporting an ORR of 71%, including 53% with CR and 18% with PR. The remaining 29% did not respond to the treatment (88). Funke et al. retrospectively analyzed 34 patients with grades II–IV SR-GVHD treated with basiliximab. The complete response rate was 84% in patients with skin involvement (27/32), 48% in those with GI GVHD (12/25), and 26% in patients with liver involvement (6/23) (89).

Pourhassan et al. studied dual cytokine blockade with basiliximab plus infliximab in 60 SR-GVHD patients. The ORRs at days 7, 14, and 28 were 28.3%, 38.3%, and 38.3%, respectively. A prior ruxolitinib exposure was associated with lower response rates (90). Nadeau et al. retrospectively studied 21 patients with severe SR-GVHD treated with basiliximab plus infliximab. The ORR was 76%, with 43% CR, achieved at a median of 21 days from treatment initiation. Notably, all survivors eventually developed cGVHD (91).

In a study by Liu et al., 129 patients with SR-GVHD were randomized to receive either basiliximab or ruxolitinib. On day 28, the ORR was significantly higher in the ruxolitinib group (72.8% vs. 54.2%, *p* = 0.031), as was the CR rate (58.0% vs. 35.4%, *p* = 0.013). Additionally, ruxolitinib was associated with a lower 1-year cumulative incidence of cGVHD (29.6% vs. 43.8%, *p* = 0.021) and non-relapse mortality (16.1% vs. 37.5%, *p* = 0.005) (92). Gao et al. assessed the combination of vedolizumab and basiliximab in 28 patients with gastrointestinal SR-GVHD. The ORR at day 28 was 75.0%, with 18 patients achieving a complete response (93).

### 5.9 Belumosudil

Rho-associated coiled-coil-containing protein kinase 2 (ROCK2) is a key regulator of immune response and fibrosis signaling pathways, including Th17-mediated pro-inflammatory mechanisms (94). Belumosudil is an oral selective ROCK2 inhibitor approved for cGVHD in patients ≥12 years who did not respond to at least two prior lines of therapy. ROCK2 inhibition by belumosudil exerts immunomodulatory and antifibrotic effects, reducing the inflammatory and fibrotic features of cGVHD (94).

In a prospective study, Inamoto et al. evaluated the efficacy of belumosudil (200 mg once daily) in 21 patients with steroid-dependent or steroid-refractory cGVHD (94).

The best ORR at 24 weeks after enrollment of the last patient was 85.7%. Notably, the lower bound of the 95% confidence interval exceeded the predefined 25% threshold, demonstrating meaningful clinical activity (94). Cutler et al. conducted a phase II randomized, multicenter registration study evaluating belumosudil administered at 200 mg once daily (*n* = 66) and 200 mg twice daily (*n* = 66) in patients with cGVHD who had received two to five prior lines of therapy. The best ORRs were 74% (once daily) and 77% (twice daily), with consistently high rates across all subgroups. Notably, complete responses were observed in all affected organs (95).

Retrospective studies further supported the efficacy of belumosudil. Caputo et al. analyzed 20 patients with steroid-refractory cGVHD who received combination therapy with ruxolitinib and belumosudil. The ORR defined as either complete response or partial response at any time was 55% (96). Similarly, Michonneau et al. conducted a retrospective study involving 68 patients with steroid-refractory cGVHD who received belumosudil monotherapy. The best achieved ORR was 57.3%, including 14.7% complete responses and 42.6% partial responses (97).

These findings underscore belumosudil as a promising therapeutic option for patients with cGVHD refractory to standard immunosuppressive therapies, owing to its dual antifibrotic and immunomodulatory mechanisms of action.

### 5.10 Abatacept

Abatacept is a recombinant fusion protein consisting of the extracellular domain of CTLA-4 linked to a modified Fc fragment of human IgG1 that lacks effector functions. Its mechanism of action involves blocking the interaction between CD28 on T cells and CD80/CD86 on antigen-presenting cells, thereby attenuating CD28-mediated T-cell activation (98).

Koshy et al. conducted a phase II clinical trial assessing the efficacy of abatacept in a cohort of 35 patients with steroid-refractory cGVHD. The study reported an ORR of 58%, with all responding patients achieving partial responses (98).

Nahas et al. conducted a phase I clinical trial evaluating the safety and efficacy of abatacept in 16 patients with steroid−refractory cGVHD. Among evaluable patients, seven (44%) achieved a clinical PR, defined as improvement in at least two organ systems according to the 2011 NIH consensus criteria. Importantly, treatment with abatacept led to a significant 51.3% reduction in mean prednisone dose among the responders, from 27 mg at baseline to 14 mg at 1 month after the sixth dose (*p* = 0.01) (99).

Abatacept has been approved by the FDA for the prevention of GVHD.

### 5.11 Axatilimab

Axatilimab is a high-affinity, humanized IgG4 monoclonal antibody that targets the ligand-binding domain of colony-stimulating factor 1 receptor (CSF-1R), thereby inhibiting both colony-stimulating factor 1 (CSF-1) and interleukin-34 (IL-34). By inhibiting ligand-induced monocyte activation without triggering receptor internalization, axatilimab exhibits potent immunomodulatory activity with therapeutic potential in conditions such as cGVHD (100).

In a phase I/II study, Kitko et al. evaluated the efficacy of axatilimab in 40 patients with active cGVHD following the failure of at least two prior lines of systemic therapy (100). The ORR within the first six treatment cycles was 82%, with clinical improvement observed across all organ systems involved. Additionally, 58% of the patients reported significant symptom relief, as measured by the Lee Symptom Scale (100). Wolff et al. conducted a randomized phase II trial in a cohort of 241 patients with recurrent or refractory cGVHD who were treated with varying doses of axatilimab. The ORR was 74% in the 0.3-mg/kg group, 67% in the 1-mg/kg group, and 50% in the 3-mg/kg group. These findings indicate dose-dependent efficacy, with an inverse relationship between dose and tolerability or response in this study population (101).

## 6 Conclusions

Among the gastrointestinal GVHD biomarkers investigated to date, sST2 and REG3α have emerged as the most promising and are already implemented for clinical use within the MAGIC algorithm in certain clinical centers. Recent studies have also highlighted the potential diagnostic and prognostic value of TIM3, TNFR1, GLP2, sIL-2Rα, amphiregulin, and IL-6, which may provide complementary information on the underlying immunopathology of GVHD. However, further confirmatory studies are needed to establish the optimal timing for sample collection and relevant cutoff values. Another limitation is the potential confounding impact of other post-alloHCT complications on biomarker concentrations. To date, studies evaluating these biomarkers have been limited to relatively small patient cohorts and remain less extensive than those focusing on REG3α and sST2. Integrating multiple biomarkers reflecting different pathogenic pathways could improve risk stratification and guide personalized therapeutic approaches in the future. Although novel therapeutic approaches targeting key pathogenic pathways are being actively explored, most studies are limited by small patient cohorts and heterogeneous study designs. Continued research is essential, both for the identification and validation of novel biomarkers and for the development of innovative therapeutic strategies.
